# Supplementary material for: A quantitative redox proteome of the human muscle response to exercise
Source: bioRxiv. 2026 Sep 17:2026.09.15.751463. Preprint. [Version 1] doi: 10.64898/2026.09.15.751463 (PMC13596570; doi:10.64898/2026.09.15.751463)
Supplement: Supplementary file 1 [file NIHPP2026.09.15.751463v1-supplement-6.pdf]

bioRxiv preprint doi: https://doi.org/10.64898/2026.09.15.751463; this version posted September 17, 2026. The copyright holder for this preprint (which was not certified by peer review) is the author/funder, who has granted bioRxiv a license to display the preprint in perpetuity. It is made available under a CC-BY 4.0 International license.

SUPPLEMENTARY MATERIAL

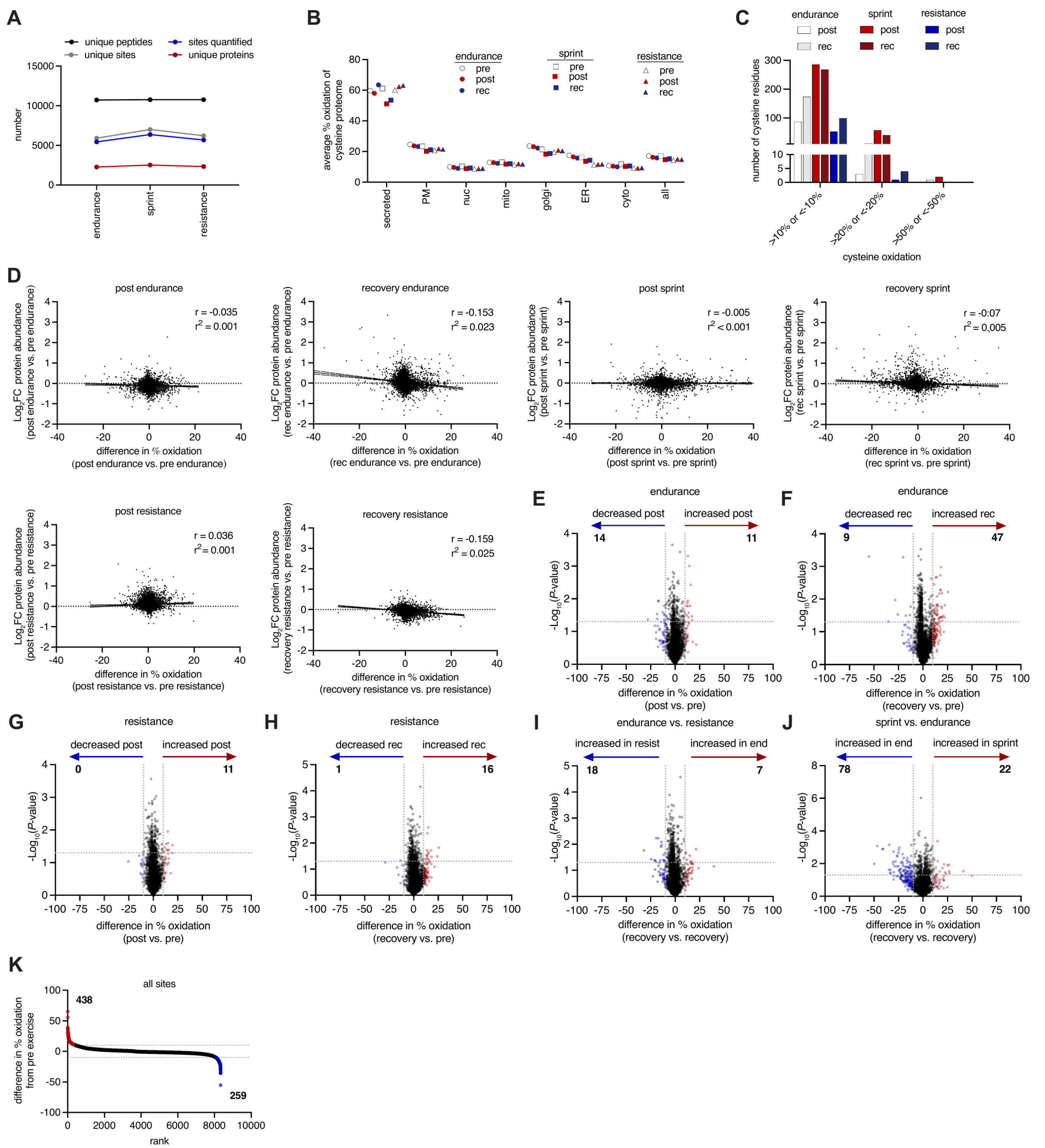

Figure S1. | **OxiMuscle Data Characteristics.**

bioRxiv preprint doi: https://doi.org/10.64898/2026.09.15.751463; this version posted September 17, 2026. The copyright holder for this preprint (which was not certified by peer review) is the author/funder, who has granted bioRxiv a license to display the preprint in perpetuity. It is made available under a CC-BY 4.0 International license.

(A) Numbers of unique peptides, cysteine sites, cysteine sites quantified and unique proteins identified. [Values are sums across n = 8 biological replicates per exercise condition.]

(B) Average percent oxidation distribution of the cysteine proteome within subcellular locations for each exercise type.

(C) Proportion of cysteines with a shift in oxidation state from respective pre-exercise of > 10% or < -10%, > 20% or < -20%, and > 50% or < -50%, in each exercise type, post and in recovery.

(D) Correlation between $\log_2$ fold change in total protein abundance and percent shift in cysteine oxidation state, both calculated relative to respective pre-exercise levels at post-exercise and recovery timepoints, across all exercise types. Total cysteine channel was used to represent changes in total protein abundance measured.

(E-F) Pairwise comparison of cysteine modification state between endurance post and recovery vs. the pre-exercise proteome. [n = 8 biological replicates per condition. Differentially modified cysteines were identified by two-sided paired Student's t-test; highlighted/colored cysteines indicate sites reaching |Δ% modification| > 10%.]

(G-H) Pairwise comparison of cysteine modification state between resistance post and recovery vs. the pre-exercise proteome. [n = 8 biological replicates per condition; statistical test and thresholds as in (E-F).]

(I) Pairwise comparison of cysteine modification state between endurance recovery vs. resistance recovery proteome. [n = 8 biological replicates per condition; statistical test and thresholds as in (E-F).]

(J) Pairwise comparison of cysteine modification state between sprint recovery vs. endurance recovery proteome. [n = 8 biological replicates per condition; statistical test and thresholds as in (E-F).]

(K) Ranking all cysteine sites quantified by percent shift from respective pre-exercise.

All analyses use n = 8 biological replicates (participants) per exercise condition. Statistical significance of cysteine oxidation changes was assessed by two-sided paired Student's t-test; cysteines passing $P < 0.05$ are annotated as significantly modified, cysteines passing $P < 0.05$ and |Δ% modification| > 10% are annotated as highly modified.

bioRxiv preprint doi: https://doi.org/10.64898/2026.09.15.751463; this version posted September 17, 2026. The copyright holder for this preprint (which was not certified by peer review) is the author/funder, who has granted bioRxiv a license to display the preprint in perpetuity. It is made available under a CC-BY 4.0 International license.

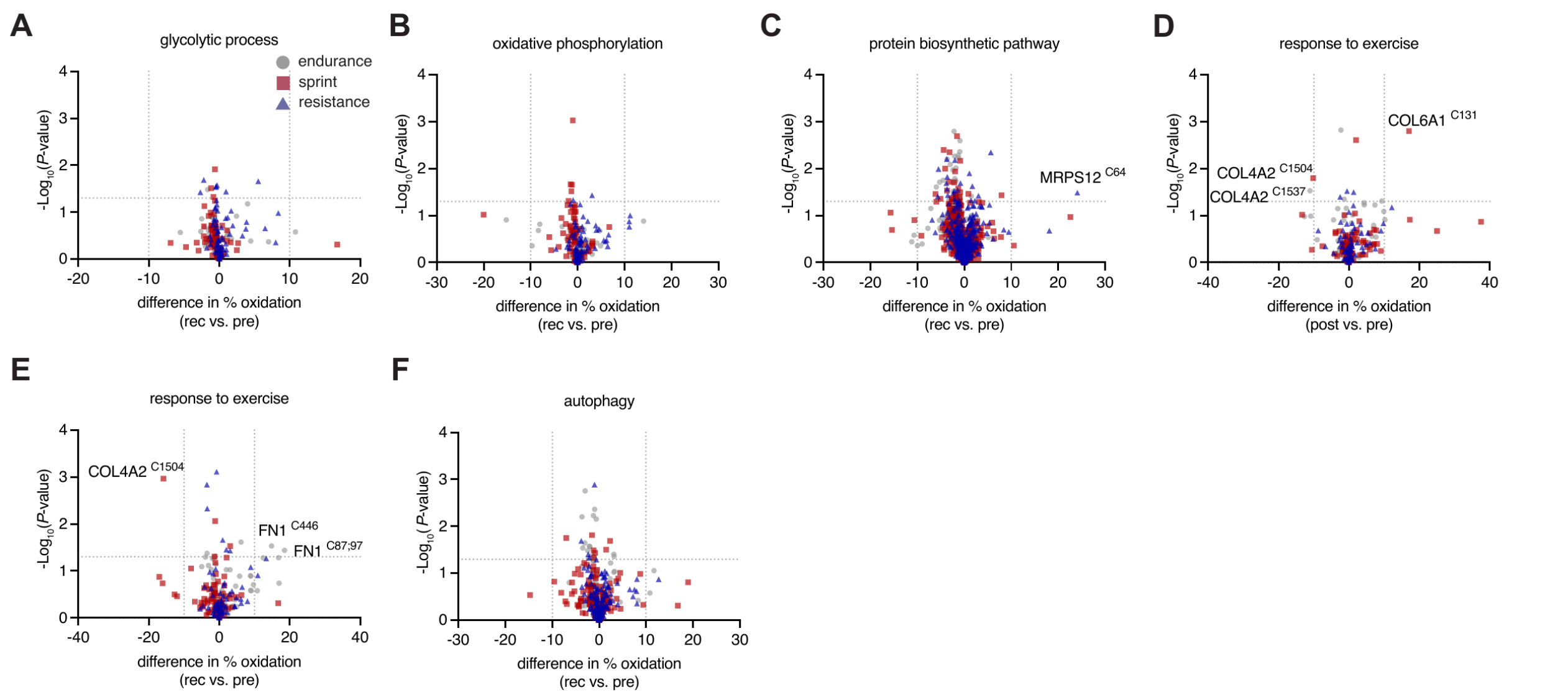

Figure S2. | Exercise-modality-specific Cysteine Oxidation Changes in Proteins Across Key Metabolic and Structural Pathways.

bioRxiv preprint doi: https://doi.org/10.64898/2026.09.15.751463; this version posted September 17, 2026. The copyright holder for this preprint (which was not certified by peer review) is the author/funder, who has granted bioRxiv a license to display the preprint in perpetuity. It is made available under a CC-BY 4.0 International license.

(A) Pairwise comparison of cysteine modification state recovery vs. pre-exercise proteome in each exercise type and filtered for proteins in the glycolytic process GO pathway. [n = 8 biological replicates per exercise condition; highlighted cysteines reach $P < 0.05$ (two-sided paired Student's t-test) and $|\Delta\%$ modification$| > 10\%$.]

(B) Pairwise comparison of cysteine modification state recovery vs. pre-exercise proteome in each exercise type and filtered for proteins in the oxidative phosphorylation GO pathway. [n = 8 biological replicates per condition; statistical test and thresholds as in (A).]

(C) Pairwise comparison of cysteine modification state recovery vs. pre-exercise proteome in each exercise type and filtered for proteins in the protein biosynthetic GO pathway. MRPS12, small ribosomal subunit protein uS12m. [n = 8 biological replicates per condition; statistical test and thresholds as in (A).]

(D) Pairwise comparison of cysteine modification state post vs. pre-exercise proteome in each exercise type and filtered for proteins in the response to exercise GO pathway. FN1, fibronectin; COL4A2, collagen alpha-2(IV) chain. [n = 8 biological replicates per condition; statistical test and thresholds as in (A).]

(E) Pairwise comparison of cysteine modification state recovery vs. pre-exercise proteome in each exercise type and filtered for proteins in the response to exercise GO pathway. COL6A1, collagen alpha-1(VI) chain. [n = 8 biological replicates per condition; statistical test and thresholds as in (A).]

(F) Pairwise comparison of cysteine modification state recovery vs. pre-exercise proteome in each exercise type and filtered for proteins in the autophagy GO pathway. [n = 8 biological replicates per condition; statistical test and thresholds as in (A).]

All analyses use n = 8 biological replicates (participants) per exercise condition. Highlighted cysteines pass $P < 0.05$ (two-sided paired Student's t-tests) and $|\Delta\%$ modification$| > 10\%$.

bioRxiv preprint doi: https://doi.org/10.64898/2026.09.15.751463; this version posted September 17, 2026. The copyright holder for this preprint (which was not certified by peer review) is the author/funder, who has granted bioRxiv a license to display the preprint in perpetuity. It is made available under a CC-BY 4.0 International license.

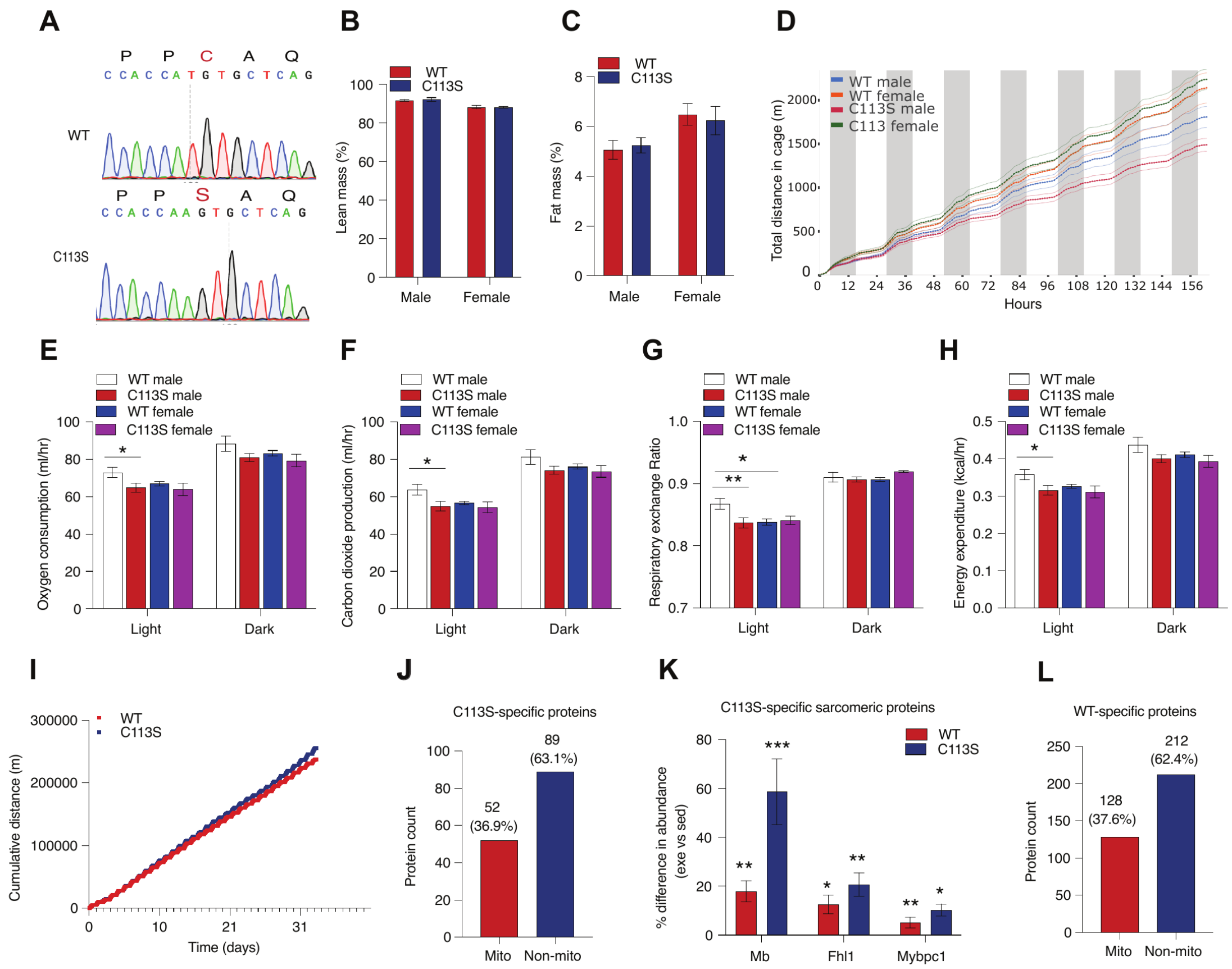

Figure S3. | Metabolic Characterization of p62 C113S Mice at Baseline and Proteome Remodeling After Exercise.
(A) Sanger sequencing chromatograms of C2C12 cells confirming the CRISPR/Cas9-mediated TGT→AGT (C113S) substitution. [Chromatogram representative of n = 3 independently isolated clonal lines.]

bioRxiv preprint doi: https://doi.org/10.64898/2026.09.15.751463; this version posted September 17, 2026. The copyright holder for this preprint (which was not certified by peer review) is the author/funder, who has granted bioRxiv a license to display the preprint in perpetuity. It is made available under a CC-BY 4.0 International license.

(B–C) Body composition analysis showing lean mass and fat mass percentages in male and female WT and p62 C113S mice measured by EchoMRI. [n = 9–11 per group.]

(D) Total distance in cage (m) assessed under basal conditions. [n = 4–7 per group.]

(E–H) Indirect calorimetry measurements including oxygen consumption (VO_2), carbon dioxide production (VCO_2), respiratory exchange ratio (RER), and energy expenditure in WT and p62 C113S mice. [n = 4–7 per group.]

(I) Cumulative running distance (m) of WT and C113S mice during voluntary wheel running. [n = 8 per group.]

(J) Proportion of mitochondrial and non-mitochondrial proteins among C113S-specific exercise-responsive proteins. [n = 9–11 per group.]

(K) Differential regulation of sarcomeric proteins between WT and p62 C113S mice following exercise. [n = 9–11 per group.]

(L) Proportion of mitochondrial and non-mitochondrial proteins among WT-specific exercise-responsive proteins. [n = 9–11 per group.]

For **(B-C, E-H)**, data are presented as mean ± SEM. Statistical significance was determined using two-way ANOVA with Tukey's post-hoc test. For **(K)**, bars show mean percent difference in abundance (exercise vs. sedentary) ± SEM per genotype. Statistical significance for each protein was determined by a two-sample Welch's t-test comparing individual exercised versus sedentary mice within each genotype; *P < 0.05, **P < 0.01, ***P < 0.001.
